# Propofol directly induces caspase-1-dependent macrophage pyroptosis through the NLRP3-ASC inflammasome

**DOI:** 10.1038/s41419-019-1761-4

**Published:** 2019-07-17

**Authors:** Lingbin Sun, Wei Ma, Wenli Gao, Yanmei Xing, Lixin Chen, Zhengyuan Xia, Zhongjun Zhang, Zhongliang Dai

**Affiliations:** 1 The Department of Anesthesiology, The Second Clinical Medical College (Shenzhen People’s Hospital), Jinan University, No. 1017 Dongmen North Road, Shenzhen, People’s Republic of China; 2 Integrated Chinese and Western Medicine Postdoctoral Research Station, Jinan University, Shipai, Guangzhou People’s Republic of China; 3 Translational Medicine Collaorative Innovation Center, The Second Clinical Medical College (Shenzhen People’s Hospital), Jinan University, No. 1017 Dongmen North Road, Shenzhen, People’s Republic of China; 4 Department of Pharmacology, Medical College, Jinan University, Shipai, Guangzhou, People’s Republic of China; 5 Department of Anesthesiology, Li Ka Shing Faculty of Medicine, The University of Hong Kong, Pokfulam, Hong Kong SAR People’s Republic of China

**Keywords:** Inflammasome, Inflammasome

## Abstract

Propofol infusion syndrome (PRIS) is an uncommon life-threatening complication observed most often in patients receiving high-dose propofol. High-dose propofol treatment with a prolonged duration can damage the immune system. However, the associated molecular mechanisms remain unclear. An increasing number of clinical and experimental observations have demonstrated that tissue-resident macrophages play a critical role in immune regulation during anaesthesia and procedural sedation. Since the inflammatory response is essential for mediating propofol-induced cell death and proinflammatory reactions, we hypothesised that propofol overdose induces macrophage pyroptosis through inflammasomes. Using primary cultured bone marrow-derived macrophages, murine macrophage cell lines (RAW264.7, RAW-asc and J774) and a mouse model, we investigated the role of NLRP3 inflammasome activation and secondary pyroptosis in propofol-induced cell death. We found that high-dose propofol strongly cleaved caspase-1 but not caspase-11 and biosynthesis of downstream interleukin (IL)-1β and IL-18. Inhibition of caspase-1 activity blocks IL-1β production. Moreover, NLRP3 deletion moderately suppressed cleaved caspase-1 as well as the proportion of pyroptosis, while levels of AIM2 were increased, triggering a compensatory pathway to pyroptosis in NLRP3^-/-^ macrophages. Here, we show that propofol-induced mitochondrial reactive oxygen species (ROS) can trigger NLRP3 inflammasome activation. Furthermore, apoptosis-associated speck-like protein (ASC) was found to mediate NLRP3 and AIM2 signalling and contribute to propofol-induced macrophage pyroptosis. In addition, our work shows that propofol-induced apoptotic initiator caspase (caspase-9) subsequently cleaved effector caspases (caspase-3 and 7), indicating that both apoptotic and pyroptotic cellular death pathways are activated after propofol exposure. Our studies suggest, for the first time, that propofol-induced pyroptosis might be restricted to macrophage through an NLRP3/ASC/caspase-1 pathway, which provides potential targets for limiting adverse reactions during propofol application. These findings demonstrate that propofol overdose can trigger cell death through caspase-1 activation and offer new insights into the use of anaesthetic drugs.

## Introduction

Propofol is one of the most commonly used intravenous agents for anaesthesia and procedural sedation. Although propofol has many pharmacological advantages over other anaesthetic agents, side effects, including cardiac depression, hypotension and vomiting after propofol infusion, remain inevitable in the context of abuse, even at therapeutic doses, in the absence of medical assistance^1^. Among the adverse effects of propofol, immune dysregulation has a prominent role^2,3^. Specifically, postoperative immunosuppression attributable to anaesthetics in cancer patients, for example, dysfunctions of natural killer cells and lymphocytes, accelerates metastatic progression^4^. More seriously, high-dose propofol can result in a rare complication, particularly in patients with acute neurological or inflammatory illnesses, which is known as propofol infusion syndrome (PRIS)^5–7^. An in vitro model of PRIS demonstrated that exposure to propofol suppresses macrophage phagocytosis, chemotaxis and oxidative burst^8,9^. Therefore, restricting the impacts of immune dysregulation after propofol application would be a promising strategy to reduce adverse effects. However, the mechanism underlying this immune dysregulation is still largely unknown.

Tissue-resident macrophages play a critical role in organ-specific regulation of immunity during anaesthesia and procedural sedation^10,11^. The immunomodulating effects of propofol have been previously demonstrated to impair monocyte and macrophage functions, including chemotaxis, oxidative burst, and phagocytosis^12^. Remarkably, propofol also affects proinflammatory cytokine production in macrophages. As reported, the biosynthesis of tumour necrosis factor-α (TNF-α), interleukin (IL)-1β and IL-6 in lipopolysaccharide (LPS)-activated macrophages is suppressed after treatment with propofol at a therapeutic concentration^13^. More importantly, exposure of macrophages to propofol causes inhibition of phagocytosis and apoptosis. Therefore, clarification of the mechanism by which propofol induces macrophage death would further expand our understanding of immune dysregulation by propofol.

To date, the majority of studies on anaesthesia-mediated immune suppression have focused on apoptosis^9,14^. The death of immune cells remains a subject of ongoing debate. Pyroptosis, a unique type of programmed cell death, is involved in immune regulation^15^. In contrast to apoptosis, pyroptosis is initiated by inflammasomes and plays a central role in inflammatory-associated diseases^16^. Activation of inflammasome-associated inflammatory caspases drives cleavage of gasdermin D (GSDMD), governs the cleavage and activation of caspase-1, and then results in maturation of IL-1β and IL-18^15^. Although the NLRP3 inflammasome and caspase-1 activation have been linked with anaesthesia exposure^17–21^, whether pyroptosis is involved in propofol-induced macrophage death remains largely unknown.

In this study, using bone marrow-derived macrophages **(**BMDMs**)**, murine macrophage lines and a mouse model, we report that propofol activated the immune system by selectively triggering inflammasomes in macrophages. We demonstrated that knockout of NLRP3 moderately suppressed cleaved caspase-1 and lactate dehydrogenase (LDH) levels, as well as the proportion of pyroptosis and that active caspase-1 then cleaved GSDMD to induce pyroptosis. This pathway was required for propofol-mediated inflammasome activation, as NLRP3-deficient mice did not show elevation of cytokines after propofol administration. Furthermore, we preliminarily speculated that a relationship between pyroptosis and apoptosis exists. Overall, these data demonstrated that propofol induces pyroptosis through caspase-1 and uncovered a new pathway of anaesthetic impairment of immune function.

## Results

### Propofol only induces macrophage pyroptosis

**Fig. 1:**
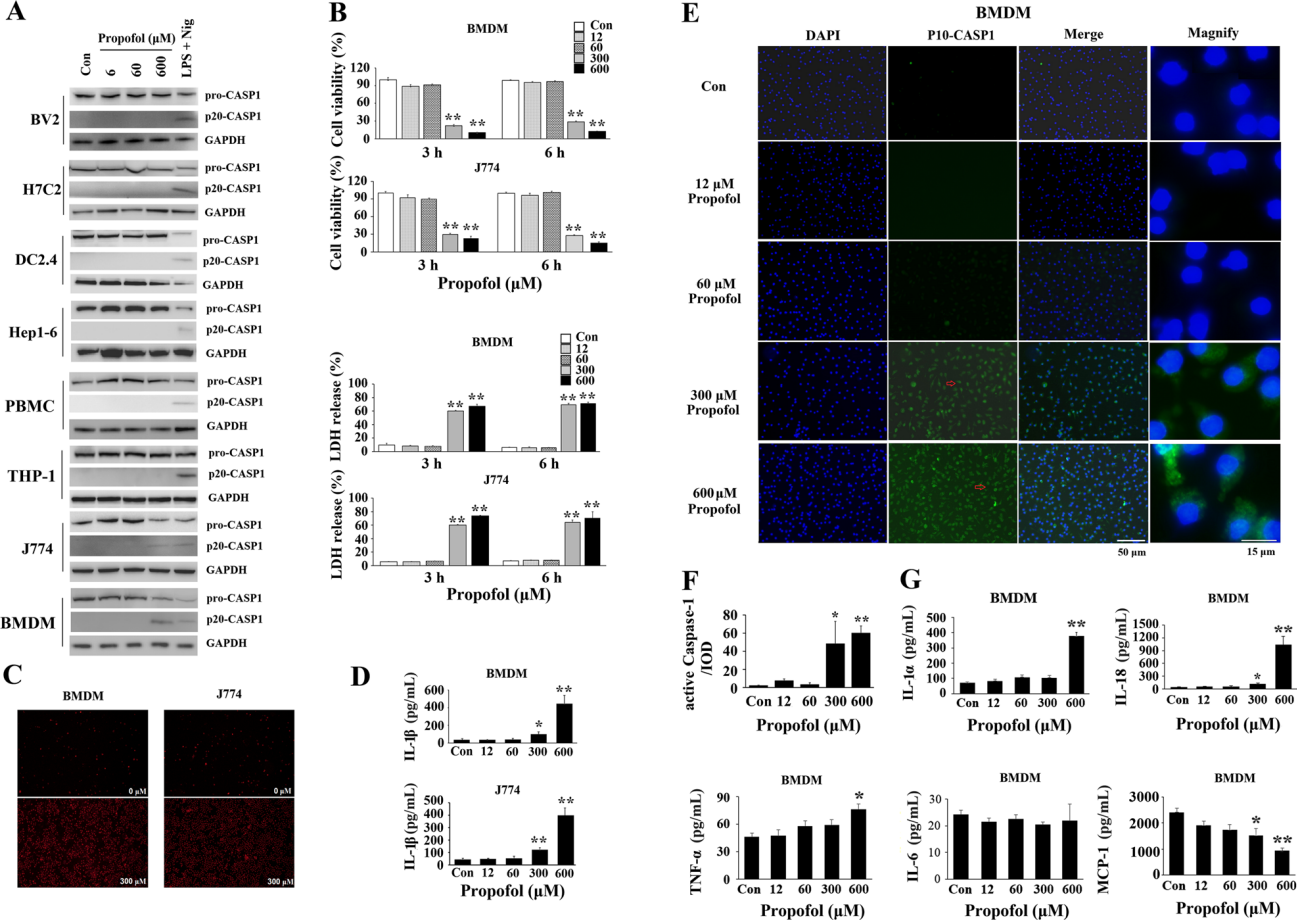
Propofol induces cleaved caspase-1 only in macrophages, but not in BV-2 microglial cells, dendritic DC2.4 cells or other cell lines. **a** Profiles of the sensitivities of various cell lines to propofol. Propofol induces cleaved caspase-1 only in BMDMs and J774 cells, but not in BV2 microglia, dendritic DC2.4 cells, PBMCs or other cell lines. **b** Propofol is cytotoxic to BMDMs and J774 cells. Cell viability and supernatant LDH activity were evaluated after exposure for 3 and 6 h in BMDMs and J774 cells. **c** Cells exposed to 300 μM propofol for 6 h were stained with PI and analysed under a microscope. **d** Following 6 h of propofol exposure, the levels of IL-1β were determined by ELISA in the supernatants. **e** Immunofluorescence staining with DAPI (blue) and of cleaved caspase-1 (green) in BMDMs after 6 h of exposure. Representative microscopy images; red arrows in the magnified image denote the fluorescence of cleaved caspase-1 (P10). Bars indicate a scale of 50 and 15 μm. **f** Cleaved caspase-1 fluorescence was further quantified and presented as IOD. **g** Propofol induced BMDM proinflammatory cytokine production after 6 h of exposure to propofol. Primary cultured BMDMs were exposed to 0, 12, 60, 300 and 600 μM propofol. After 6 h, an ELISA was performed to evaluate the levels of proinflammatory cytokines in supernatants. Concentrations of IL-1α, IL-18, TNF-α, IL-6 and MCP-1 in supernatants. The data are expressed as the mean ± SE (*n* = 3–6); *P < 0.05 and **P < 0.01 versus control; one-way ANOVA with the Duncan’s test was used for statistical analysis. IL = interleukin; TNF = tumour necrosis factor; MCP-1 = macrophage chemotactic protein-1; CASP1 = caspase-1

We first evaluated the specificity of caspase-1-induced pyroptotic cell death by treating various cell lines with propofol. Out of diverse panel of cell lines tested, only BMDMs and J774 cells released cleaved-caspase-1, suggesting that propofol’s effects might be restricted to macrophage (Fig. 1a). BV-2 microglial cells, primary human peripheral blood mononuclear cells (PBMCs) and THP-1 monocytes could not release active caspase-1 upon treatment with propofol (Fig. 1a).

Cell Counting Kit-8 (CCK-8) and LDH release assays were initially conducted to determine cell viability. As shown in Fig. 1b, propofol did not significantly change the results of the CCK-8 or LDH release assays at/below a clinically relevant concentration (60 μM). However, when the concentration of propofol was increased to 300 or 600 μM, we observed a remarkable cytotoxic effect and release of LDH after both 3- and 6-h propofol treatments. The results showed concurrent release of the cytoplasmic enzyme LDH in BMDMs after a 6-h propofol treatment (Fig. 1b). Consistent with the results above, the number of propidium iodide (PI)-positive cells was greatly increased in both BMDMs and J774 cells by 300 μM propofol (Fig. 1c). These results provide initial evidence that propofol induces macrophage cell death.

We further explored whether pyroptosis was involved in the cell death induced by propofol. The number of cleaved caspase-1-staining cells was dramatically increased in BMDMs and J774 cells exposed to 300 and 600 μM propofol for 6 h (Fig. 1e and Supplementary Fig. 1). In contrast, 12 and 60 μM propofol did not significantly induce the biosynthesis of cleaved caspase-1. Analysis of integrated optical density (IOD) showed that exposure to 300 and 600 μM propofol in BMDMs resulted in a significant increase compared to that of the control group (21.61- and 26.79-fold, respectively) (Fig. 1f). Additionally, we found that 300 and 600 μM propofol remarkably potentiated the production of IL-1β in the supernatant of BMDMs and J774 cells (Fig. 1d). To further verify the elevated level of IL-1β resulting from pyroptosis or the inflammatory response due to propofol, we also examined the expression of IL-1α, IL-18, IL-6, MCP-1 and TNF-α. Consistent with the results for IL-1β, high-dose propofol greatly enhanced the biosynthesis of IL-1α (5.17-fold in the 600 μM group) and IL-18 (3.24- and 29.63-fold in the 300 and 600 μM groups, respectively) in the supernatant of BMDMs (Fig. 1g). A slightly increased level of TNF-α (1.65-fold) compared to that of the control was noted with 600 μM treatment, while the level of MCP-1 was downregulated (Fig. 1g). These results demonstrated that the potentiated production of IL-1α, IL-1β and IL-18 was not due to a global inflammatory response but rather to the activation of caspase-1, which strongly suggested that propofol induces macrophage pyroptosis.

### Propofol induces caspase-1-dependent pyroptosis in macrophages

**Fig. 2:**
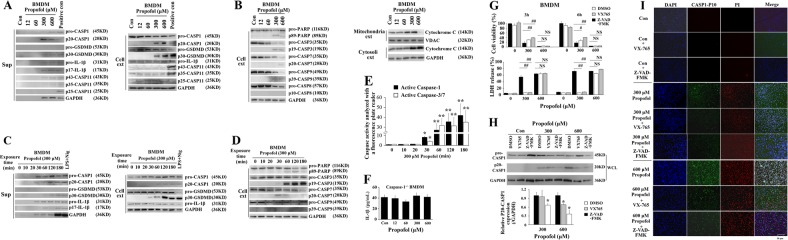
Exposure to propofol induces caspase-1-dependent pyroptosis. BMDMs were exposed to 0, 12, 60, 300 and 600 μM propofol. Following 3 h of propofol exposure, immunoblots of cell extracts and supernatants from BMDMs for proteins associated with pyroptosis (**a**) and apoptosis (**b**) were performed. **c** BMDMs were treated with propofol (300 μM) or vehicle for the indicated time periods. Immunoblots were performed to detect pyroptosis-related proteins in BMDM supernatants and cell extracts. **d** Immunoblots were performed to detect apoptosis-related proteins in cell extracts. GAPDH was the internal control. **e** BMDMs were treated with 300 μM propofol, and then, cell samples were harvested at 0, 10, 20, 30, 60, 120 and 180 min after propofol exposure. Cells were labelled with ICT’s poly-caspase inhibitor reagent. Active caspase-1 and active caspase-3/7 were analysed with a 96-well fluorescence plate reader (Spark 10 M, Tecan). **f** Following 6 h of propofol exposure, the levels of IL-1β were determined by ELISA in the supernatants of caspase-1^-/-^ BMDMs. **g** Pre-treatment (30 min) of BMDMs with VX-765 or Z-VAD-FMK blocked 300 μM propofol-induced cytotoxicity at 3 and 6 h. However, when the concentration of propofol was increased to 600 μM, caspase-1 inhibitors did not significantly change the results of the CCK-8 or LDH release assays. **h** Pre-treatment (30 min) of BMDMs with VX-765 or Z-VAD-FMK inhibited high-dose propofol-induced cleaved caspase-1. Band intensity was quantified by ImageJ software, and the values of target protein were normalised to that of GAPDH. **i** Three-hundred micromolar propofol-induced pyroptosis was inhibited by treating cells with VX-765 and Z-VAD-fmk. Immunofluorescence staining with DAPI (blue), PI (red) and of cleaved caspase-1 (green) in BMDMs after 6 h of exposure. Representative microscopy images. Bars indicate a scale of 50 μm. The results are representative of three independent experiments. Bars represent the mean ± SE (*n* = 3–5); one-way ANOVA followed by the Duncan’s test; *P < 0.05 and **P < 0.01 versus control. CASP1 = caspase-1; CASP11 = caspase-11; CASP3 = caspase-3; CASP7 = caspase-7; CASP9 = caspase-9; Sup = supernatant; Cell ext = cell extract; WCL = whole-cell lysates

In an attempt to study whether caspase-1 and/or caspase-11 mediate propofol-induced macrophage pyroptosis, immunoblotting was performed. As shown in Fig. 2a, cleaved IL-1β levels were detected in the culture supernatant after high-dose propofol exposure. Consistently, the cleaved forms of pro-caspase-1 and pro-GSDMD were detected in BMDM supernatants and cell extracts after exposure to 300 and 600 μM propofol (Fig. 2a). Similarly, 300 and 600 μM propofol induced pro-caspase-1 and pro-GSDMD cleavage in cell extracts and culture supernatants from J774 cells (Supplementary Fig. 2A). These results were comparable to those observed with LPS plus nigericin, which served as a positive control and is widely used for pyroptosis induction. In contrast, the levels of pro-caspase-11 and cleaved caspase-11 were not changed/detected (Fig. 2a). Similarly, we observed cleavage of pro-IL-1β, pro-GSDMD and pro-caspase-1 after 30 min of 300 μM propofol treatment in both supernatants and extracts from BMDMs and J774 cells (Fig. 2c and Supplementary Fig. 3A). To further verify the role of caspase-1 in propofol-induced pyroptosis, caspase-1 knockout totally eliminated the production of mature IL-1β induced by propofol (Fig. 2f). In a second approach, pre-treatment of BMDMs with VX-765 or Z-VAD-FMK blocked 300 μM propofol-induced cytotoxicity and pyroptosis (Fig. 2g–i). Altogether, the results above suggested that propofol induces caspase-1-dependent macrophage pyroptosis.

### Activation of the NLRP3/ASC inflammasome is involved in macrophage pyroptosis induced by propofol

**Fig. 3:**
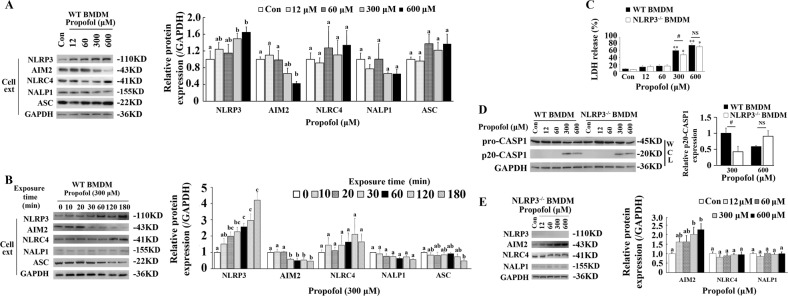
Activation of NLRP3-ASC is involved in macrophage pyroptosis induced by propofol. **a** BMDMs were exposed to 0, 12, 60, 300 and 600 μM propofol. Following 3 h of propofol exposure, immunoblots were performed to detect pyroptosis-related proteins in cell extracts. Quantitative accumulated western blot data for inflammasomes and ASC are shown. **b** Western blot analysis was used to determine the expression of inflammasomes and ASC. BMDMs were treated with propofol (300 μM) or vehicle for the indicated time periods. GAPDH was an internal control. Quantitative accumulated western blot data for inflammasomes and ASC are shown. **c** NLRP3 knockout BMDMs induce pyroptosis after exposure to propofol for 3 h. Supernatant LDH activity was assessed, and OD values at 490 nm are presented in the histogram. **d** BMDMs were exposed to 0, 12, 60, 300 and 600 μM propofol. Following 3 h of propofol exposure, western blot analysis of pro-caspase-1 and cleaved caspase-1 in WT and NLRP3^-/-^ BMDMs. Band intensity of cleaved caspase-1 was quantified by ImageJ software, and the values of target proteins were analysed. **e** Western blot analysis of inflammasomes in NLRP3^-/-^ BMDMs; band intensity was quantified by ImageJ software, and the values of target proteins were normalised to that of GAPDH. The results are representative of three independent experiments. The data are expressed as the mean ± SE (*n* = 3–6); *P < 0.05 and **P < 0.01 versus control; ^#^*P* < 0.05; NS, not significant; one-way ANOVA followed by the Duncan’s test. WT = wild type; WCL = whole-cell lysates; Cell ext = cell extract; CASP1 = caspase-1

Activation of AIM2 and the NLR family, including NALP1, NLRP3 and NLRC4, is believed to mediate pyroptosis through caspase-1^22^. We next explored whether AIM2 or the NLR family is involved in macrophage pyroptosis induced by propofol and found that the levels of NLRP3 were significantly upregulated by propofol in a time- and dose-dependent manner. Meanwhile, there was no change in NALP1 and NLRC4 protein expression between the control and the other groups. Interestingly, a sustained decrease in AIM2 levels compared to those of the control was observed after propofol treatment (Fig. 3a, b and Supplementary Figs. 2C and 3C).

As shown in Fig. 3c, a modest decrease in LDH levels was observed in NLRP3^-/-^ BMDMs treated with 300 μM propofol compared with those in wild-type (WT) BMDMs. Consistently, LDH release was also significantly reduced after 300 and 600 μM propofol exposure compared to that of the control in NLRP3^-/-^ BMDMs. Additionally, NLRP3^-/-^ BMDMs showed a corresponding decrease in cleaved caspase-1 in immunoblot assays after 300 μM propofol treatments compared with those in WT BMDMs, which indicated the crucial role of NLRP3 in propofol-induced macrophage pyroptosis (Fig. 3d). Notably, we found that NLRP3 knockout in BMDMs did not totally prevent the cleavage of pro-caspase-1. In addition, high-dose propofol still induced cell death in NLRP3^-/-^ BMDMs, which implied that compensatory signals were involved after NLRP3 deletion. To validate this hypothesis, we measured AIM2, NALP1 and NLRC4 expression in NLRP3^-/-^ BMDMs and found that the levels of AIM2 were greatly upregulated by propofol after NLRP3 knockout (Fig. 3e). Analysis of IOD showed that exposure to 300 and 600 μM propofol in BMDMs resulted in significant increases compared to that in the control group. Collectively, these results suggest that NLRP3 is required for macrophage pyroptosis induced by propofol and that a possible compensatory effect by AIM2 after NLRP3 deletion exists.

**Fig. 4:**
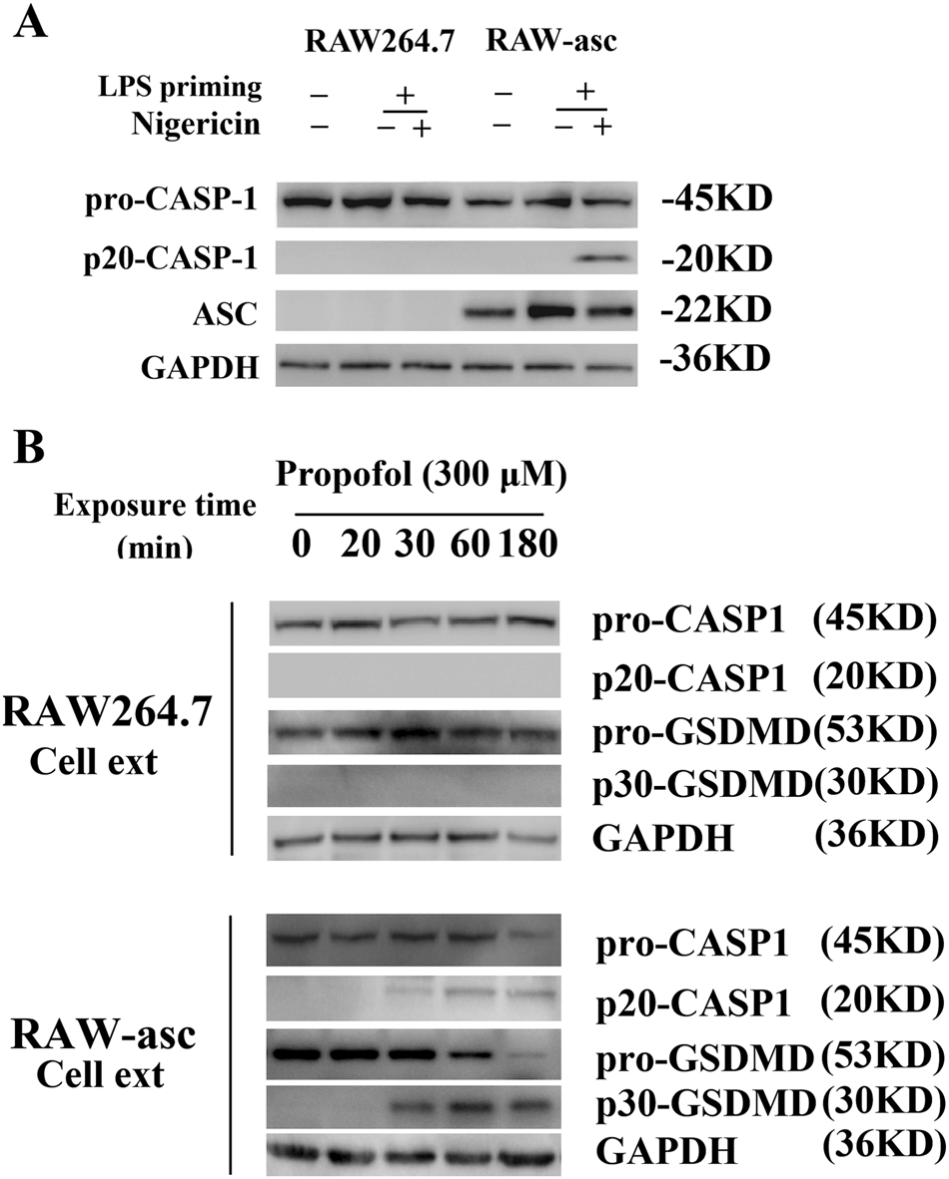
ASC mediates propofol-induced pyroptosis. **a** Processing of caspase-1 in RAW264.7 and RAW-asc cell lines treated with LPS plus nigericin. The cells were primed with LPS for 4 h and then treated with nigericin for 2 h. **b** Exposure of RAW-asc cells to 300 μM propofol for 20, 30, 60 and 180 min induced pyroptosis through caspase-1 cleavage of GSDMD. Pyroptotic caspases were not activated in RAW264.7 cells. The results are representative of three independent experiments. Cell ext = cell extract; CASP1 = caspase-1

Downstream of NLRs, ASC plays an important role in the assembly of inflammasomes and the recruitment of caspase-1^23^. To investigate the potential role of ASC in propofol-induced pyroptosis, another macrophage cell line, RAW264.7, was used, as ASC is deleted in this cell line. As shown in Fig. 4a, no ASC expression was observed in RAW264.7 cells. Additionally, no cleaved caspase-1 was detected in immunoblotting analysis after administration of LPS and nigericin. Similarly, 300 μM propofol did not induce the cleavage of caspase-1 and GSDMD even after 3 h of treatment (Fig. 4b).

The plasmid/virus inserted complementary DNA of mouse ASC was delivered into RAW264.7 cells to produce a stable ASC-expressing line (RAW-asc) for screening. RAW-asc cells responded to both LPS/nigericin and propofol, leading to the cleavage of caspase-1 and GSDMD (Fig. 4a, b). Altogether, the results above indicated that ASC mediates NLRP3 signalling and further contributes to macrophage pyroptosis induced by propofol.

### Propofol-mediated mitochondrial damage in BMDMs

**Fig. 5:**
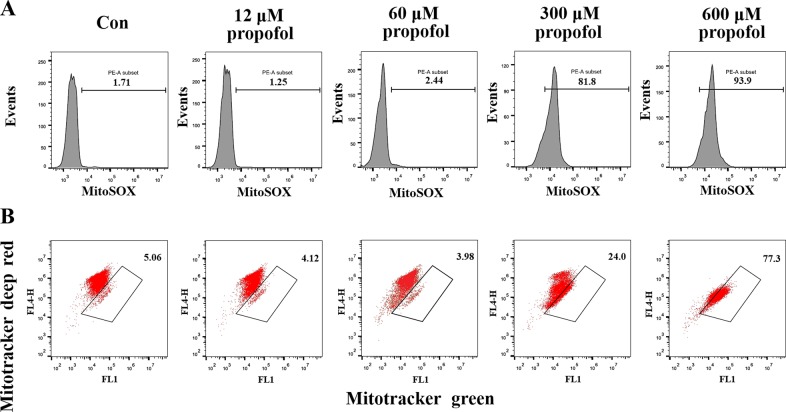
Propofol induces mitochondrial damage. BMDMs were stimulated with propofol for 20 min and then stained with MitoSOX (**a**) or MitoTracker Green and MitoTracker Deep Red (**b**) for 30 min and analysed by flow cytometry

Recent research suggests that mitochondrial damage results in NLRP3 inflammasome activation^24^. Here, we investigated a possible implication of propofol-induced mitochondrial damage in inflammasome activation. BMDMs cultured in six-well plates were treated with propofol for 20 min. Importantly, 300 and 600 μM propofol treatment of BMDMs triggered rapid changes in mitochondrial reactive oxygen species (ROS) production and membrane potential (ΔΨ_m_), suggesting the occurrence of propofol-induced mitochondrial damage (<30 min) prior to NLRP3 inflammasome activation and cleavage of caspase-1 (Fig. 5a, b). These data are compatible with the notion that propofol-induced mitochondrial ROS can lead to NLRP3 inflammasome activation.

### Propofol induced mitochondria-dependent apoptosis in caspase-1^-/-^ BMDMs

Dysregulation of mitochondria is also involved in propofol-induced cell apoptosis. To investigate the molecular mechanisms of propofol overdose-induced apoptosis, the concentration gradient and time kinetics of the propofol-induced caspase cascade in BMDMs and J774 cells were shown by immunoblotting analysis (Fig. 2b, d and Supplementary Figs. 2B and 3B). As shown in Fig. 2b and Supplementary Fig. 2B, the levels of cleaved caspase-3, caspase-7, caspase-9 and PARP and release of cytochrome C were induced by 300 and 600 μM propofol. In addition, apoptotic cellular death pathways were activated after 30 min of propofol treatment (Fig. 2d and Supplementary Fig. 3B). In summary, our results indicated that propofol-induced apoptosis was not mediated through extrinsic pathways. Propofol induced apoptosis through a mitochondrion-dependent pathway. Furthermore, both caspase-1 and caspase-3/7 activity in BMDMs showed a significant increase after 30 min of 300 μM propofol treatment. However, no significant difference was observed between active caspase-1 and caspase-3/7 after exposure to propofol, with an approximately equal amount of apoptotic cell death and pyroptotic cell death (Fig. 2e).

**Fig. 6:**
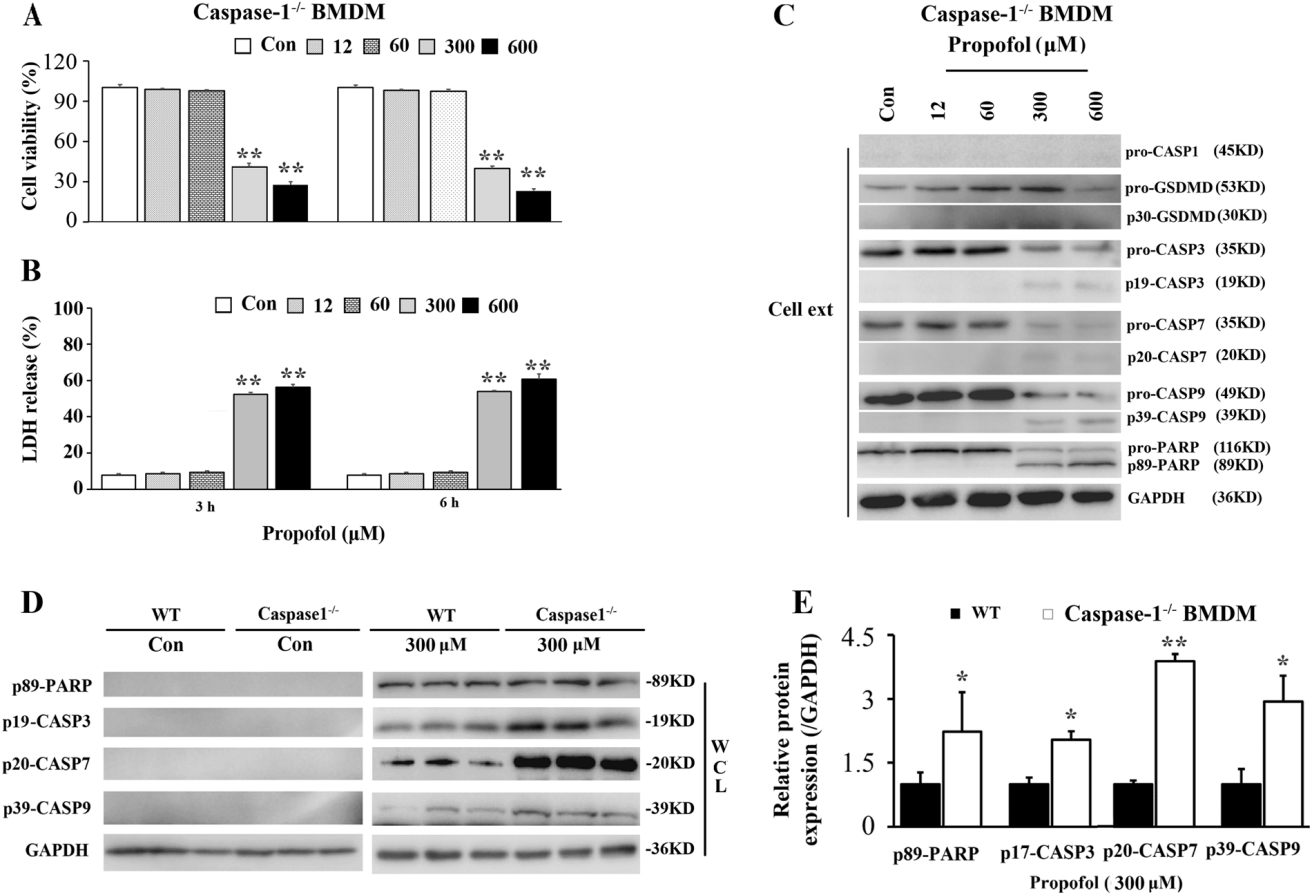
Propofol-induced cell death is attenuated in caspase-1^-/-^ BMDMs. Caspase-1^-/-^ BMDMs were exposed to 0, 12, 60, 300 and 600 μM propofol. **a** Following 3 and 6 h of propofol exposure, cell viability was evaluated. **b** Supernatant LDH activity was measured in cultured caspase-1^-/-^ BMDMs, and OD values at 490 nm are presented. **c** Immunoblots were performed to detect apoptosis-related proteins in cell extracts. **d** Knockout of caspase-1 aggravated propofol-induced apoptosis in BMDMs. Caspase-1^-/-^ BMDMs were treated with propofol (300 μM) or vehicle. Following 3 h of propofol exposure, western blot analysis of apoptosis-related proteins in caspase-1^-/-^ BMDMs was performed. **e** Band intensity was quantified by ImageJ software, and the values of target proteins were normalised to that of GAPDH. The results were representative of three independent experiments; *P < 0.05 and **P < 0.01 versus control; one-way ANOVA followed by the Duncan’s test. CASP1 = caspase-1; CASP3 = caspase-3; CASP7 = caspase-7; CASP9 = caspase-9; Cell ext = cell extract; WCL = whole-cell lysates

To further validate the significance of caspase-1 in propofol-induced pyroptosis, we investigated the effect of caspase-1 silencing on mitochondrion-mediated apoptosis in macrophages. We observed a remarkable cytotoxic effect after 3 and 6 h of propofol treatment (Fig. 6a, b), while the release of IL-1β triggered by propofol was abrogated in caspase-1^-/-^ BMDMs (Fig. 2f). In addition, knockout of caspase-1 aggravated propofol-induced apoptosis in BMDMs (Fig. 6c). We found that knockout of caspase-1 had a significant impact on the levels of cleaved caspase-3, caspase-7, caspase-9 and PARP compared to those in WT BMDMs (Fig. 6d, e). We speculated that macrophages lacking caspase-1 are susceptible to propofol-induced apoptosis and there is cross talk between pyroptosis and apoptosis.

### Propofol induces pyroptosis in splenic macrophages

Tissue-resident macrophages are a heterogeneous population of immune cells that fulfil tissue-specific functions. Since splenic macrophages play an important role in host defence by bridging the innate and adaptive immune systems, we examined whether propofol induces pyroptosis in splenic macrophages. Two-month-old mice were intraperitoneally administered propofol at 0, 50, or 100 mg/kg/d for 7 consecutive days. After 7 days of exposure, splenic macrophages were isolated as described previously^25^.

**Fig. 7:**
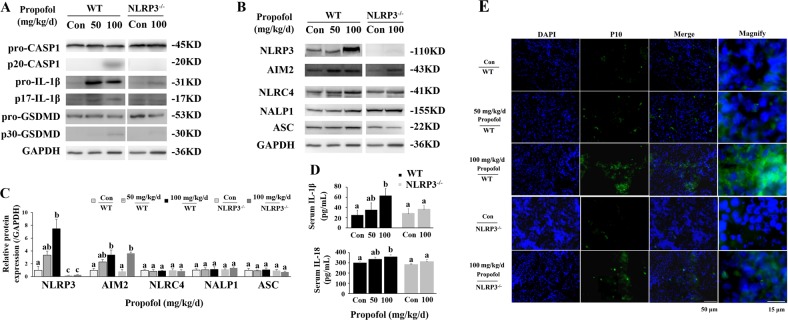
Knockout of NLRP3 alleviates propofol-induced pyroptosis in splenic macrophages. **a** Western blot analysis of pyroptosis-related proteins. **b** Western blot analysis of inflammasome-related proteins and ASC. **c** Band intensity was quantified by ImageJ software, and the values of target protein were normalised to that of GAPDH. **d** ELISA analysis of serum IL-1β and IL-18. **e** Immunofluorescence staining with DAPI (blue) and of cleaved caspase-1 (green) in mouse spleens after 7 days of exposure to propofol. Representative microscopy images; Red arrows in the magnified image denote the fluorescence of cleaved caspase-1 (P10). Bars indicate 50 and 15 μm. The results are representative of three independent experiments. Bars represent the mean ± SE (*n* = 3–5). The same indexes not sharing a common letter are significantly different at *P* < 0.05 as assessed using one-way ANOVA followed by the Duncan’s test

Consistent with the results from cultured BMDMs and J774 cells, 100 mg/kg/d propofol induced the cleavage of caspase-1, GSDMD and IL-1β (Fig. 7a). Meanwhile, 100 mg/kg/d propofol induced the cleavage of caspase-3, caspase-7 and caspase-9 (Supplementary Fig. 4). Similar to in vitro observations, the serum levels of cleaved IL-1β and IL-18 were also elevated after 100 mg/kg propofol treatment compared to those of the control group (Fig. 7d). To further validate the pyroptosis induced by propofol, immunofluorescence analysis was conducted on frozen spleen slices of propofol-injected mice. Compared with mice injected with 10% intralipid, mice administered 100 mg/kg propofol exhibited an increased level of cleaved caspase-1 signal (green) in the spleen marginal zone 7 days after treatment (Fig. 7e).

### NLRP3 knockout mice are insensitive to propofol-induced pyroptosis

To study the role of NLRP3 in the regulation of propofol-induced pyroptosis in mice, the expression of NLRP3 was examined in splenic macrophages. As such, 100 mg/kg propofol greatly induced the potentiated expression of NLRP3 (7.47-fold), while there was no difference in the expression of NALP1 and NLRC4 (Fig. 7b, c). In addition, AIM2 expression was moderately upregulated after 100 mg/kg propofol administration (3.36-fold, *P* < 0.05). Consistently, after NLRP3 silencing, the cleavage of caspase-1 and GSDMD were inhibited, although the level of AIM2 expression was elevated (2.01-fold, *P* < 0.05). Additionally, the potentiated expression of cleaved IL-1β and IL-18 by propofol in serum was also inhibited in NLRP3^-/-^ mice (Fig. 7d). Importantly, the level of cleaved caspase-1 signal was reduced in the spleen marginal zone of NLRP3^-/-^ mice compared with that in WT mice (Fig. 7e). Altogether, these results demonstrated that propofol induced pyroptosis in macrophages through the NLRP3-ASC-caspase-1 pathway.

## Discussion

**Fig. 8:**
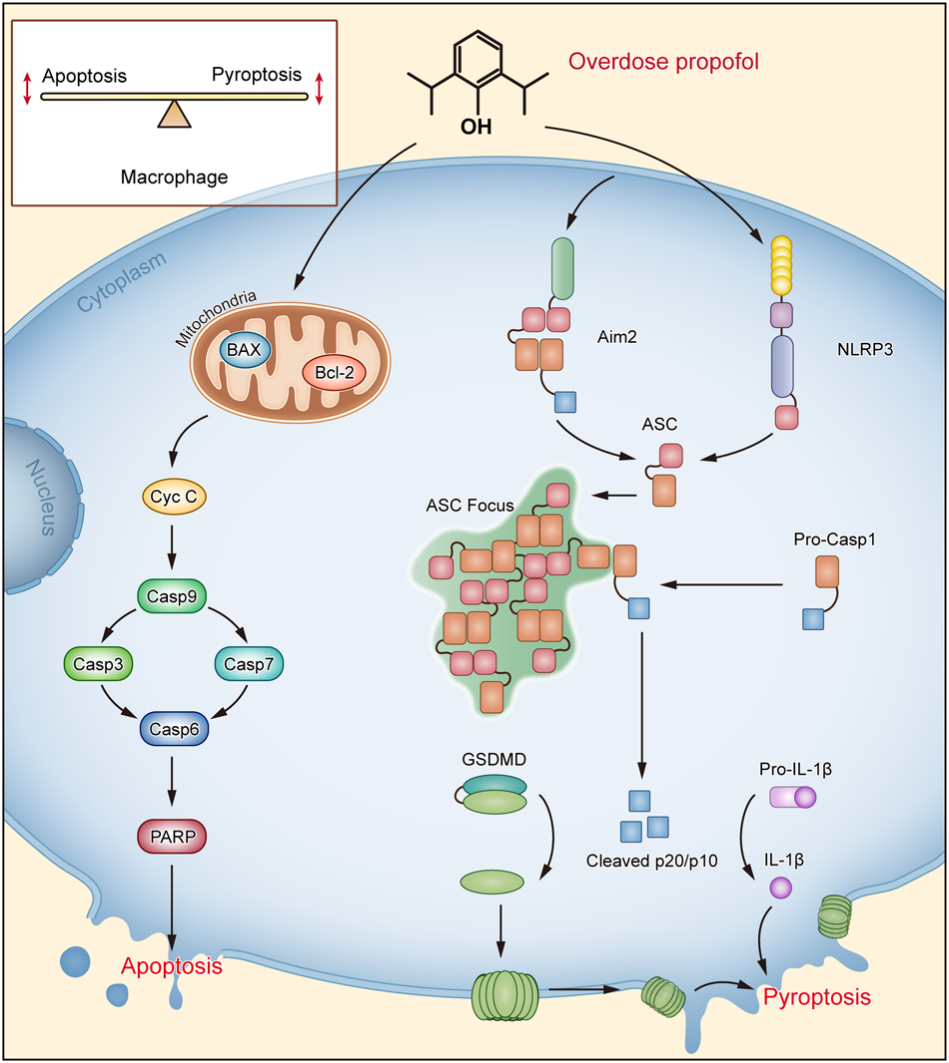
A schematic view of high-dose propofol induced mitochondrial apoptosis and caspase-1-dependent pyroptosis in macrophages. NLRP3 activation and secondary pyroptosis induce cell death after propofol exposure, as shown by cleaved-caspase-1 and cleaved IL-1β. When NLRP3 is knocked out, we speculate that the AIM2 inflammasome induces caspase-1-dependent pyroptosis. Propofol activates the proprotein form of caspase-1 in an inflammasome adaptor ASC-mediated manner via the NLRP3 and AIM2 inflammasomes. We speculate that propofol-induced mitochondrial ROS trigger NLRP3 inflammasome activation. Simultaneously, propofol also involves in the mitochondria-associated apoptosis in macrophages. Knockout of caspase-1 did not block macrophage cytotoxicity, and macrophages lacking caspase-1 are susceptible to propofol-induced apoptosis. We have provided an explanation: activation of NLRP3 is necessary and sufficient for pyroptosis in propofol-induced cell death. Both apoptotic and pyroptotic cellular death pathways were activated after propofol exposure. Thus, further uncovering the cross talk between pyroptosis and mitochondrial apoptosis will increase our understanding of anaesthetic-induced cell death

Immune dysregulation has been considered one of the key factors contributing to the adverse effects of propofol. However, the mechanism underlying this process remains largely unclear. Here, we demonstrate a novel mechanism by which propofol activation of the NLRP3/ASC/caspase-1 pathway only in macrophages leads to pyroptosis (Fig. 8). Several lines of evidence support this finding. First, propofol strongly induced the activation of caspase-1 and cell death in BMDMs and J774 cells. Second, exogenous expression of ASC in RAW264.7 cells drove pyroptosis induction by propofol. Last, and importantly, propofol activated the NLRP3 inflammasome, and NLRP3 deletion moderately inhibited pyroptosis by propofol both in vitro and in vivo, which supports the hypothesis that propofol-induced pyroptosis is not wholly NLRP3 dependent. When NLRP3 was knocked out, we speculate that the AIM2 inflammasome induced caspase-1-dependent pyroptosis. Therefore, inhibiting macrophage pyroptosis and its consequent effects to maintain macrophage functions could be an effective strategy to reduce the adverse effects of propofol.

Activation of NLRP3 plays an important role in caspase-1-dependent pyroptosis. In addition to exogenous pathogens, endogenous “danger signals” also activate the NLRP3 inflammasome, according to an increasing number of studies. There is a large amount of literature proposing a link between mitochondrial malfunction, ROS and inflammatory reactions. Specifically, mitochondrial dysfunction induces specific activation of the NLRP3 inflammasome^24,26,27^. Moreover, high-dose propofol remarkably induces mitochondrial dysfunction^12,28^. In our research, a more direct mechanism, that is, an excessive induction of mitochondrial oxidative stress, is likely to activate the NLRP3 inflammasome signalling pathway. Therefore, further studies are needed to investigate whether propofol-induced mitochondrial ROS are responsible for the activation of both the inflammasome and apoptosome, which can probably help us understand the role of mitochondria in macrophage cell death.

An important implication of our findings is that AIM2 acts as a compensatory factor mediating macrophage pyroptosis after NLRP3 deletion. As a cytosolic dsDNA sensor, AIM2 interacts with ASC and recruits pro-caspase-1 to the inflammasome, which leads to the autoactivation of caspase-1 and further biosynthesis of IL-1β^29^. Here, NLRP3 knockout in turn mediated the elevated expression of AIM2 by propofol. Furthermore, the cleavage of caspase-1 was partially retained. Therefore, these findings suggest that it would be difficult to reverse cell fate once it was determined, especially in conditions with multiple compensatory pathways. An early intervention with new targets upstream of NLRP3 would be more promising to inhibit the pyroptosis of macrophages.

Since NLRP3 and AIM2 inflammasomes are essential for propofol-induced pyroptosis, it is likely that ASC is essential for propofol-induced pyroptosis. Notably, propofol resulted in significant cleavage of GSDMD and caspase-1 in RAW-asc cells but not in RAW264.7 cells. ASC plays an essential role in the assembly of the NLRP3 and AIM2 inflammasomes, bridging pro-caspase-1 and pyrin-containing receptors^23^. However, the CARD-containing receptors NLRC4 and NALP1 can directly interact with pro-caspase-1 without ASC. Taken together, our data indicate that ASC mediates propofol-induced pyroptosis and that ASC is essential for pyroptosis induced by the NLRP3 and AIM2 inflammasomes, consistent with previous results demonstrating that propofol-induced NLRP3^-/-^ BMDM pyroptosis was mediated by the AIM2 inflammasome.

Evidence has shown that apoptosis, another form of cell death, is also responsible for propofol-induced cell death in vitro^9,30,31^. In the present study, we observed the cleavage of caspase-3/7 and the release of cytochrome C. Additionally, apoptosis-induced cell death in BMDMs was comparable to pyroptosis, indicating its important role in propofol-mediated macrophage dysfunction. However, in addition to caspase activation and cell damage, pyroptosis is also associated with significant release of IL-1β and IL-18, which in turn promote the inflammatory process. Specifically, alveolar macrophages treated with high-dose propofol undergo inflammatory reactions, including aggregation, phagocytosis, and release of cytokines^32^. However, an ex vivo study revealed that propofol at a therapeutic concentration had anti-inflammatory and antioxidative effects on the biosynthesis of TNF-α, IL-1β, IL-6 and NO in LPS-activated macrophages^13^. Additional physiological evidence has revealed that propofol might relieve the inflammatory response and attenuate brain injury by inhibiting ROS and depressing NLRP3 inflammasome activation and proinflammatory cytokine maturation^18^. The immunomodulation by propofol is currently purposed as a mechanism for its additional pharmacologic actions. Studies showed that the mechanism for therapeutic concentrations of propofol-induced anti-inflammatory effect is generally focused on nuclear factor-kappa B activation^13^. In this study, we found that high-dose propofol induces macrophage pyroptosis through inflammasomes and further biosynthesis of IL-1β. The major explanation for high-dose propofol-induced inflammatory response might be that propofol-induced large quantities of mitochondrial ROS are responsible for the activation of inflammasome^24^. Therefore, combined with our findings, these reports suggest that low-dose propofol would generate small quantities of ROS, prevent mitochondrial dysfunction and suppress inflammation while high doses would induce pyroptosis and inflammatory processes^18^.

At present, it is clear that pyroptosis and apoptosis are involved in propofol-induced cell death (Fig. 8). Pyroptosis occurs after activation of caspase-1. In contrast, the release of mitochondrial cytochrome C to the cytosol is considered a critical step for apoptosis. Propofol-induced apoptotic initiator caspase-9 subsequently cleaves effector caspase-3 and caspase-7. Indeed, Vince et al.^33^ found that BAX/BAK-mediated mitochondrial damage induces a parallel pathway to NLRP3 inflammasome-mediated caspase-1-dependent IL-1β maturation. Propofol-induced pyroptosis and apoptosis had similar threshold values (nearly 300 μM propofol) and initiation times (nearly 30 min). Furthermore, we showed that knockout of caspase-1 significantly increased the expression of critical proteins related to apoptosis in our in vitro cell model. Specifically, given that mitochondria play a crucial role in orchestrating activation of the NLRP3 inflammasome^34,35^, formation of the NLRP3/caspase-1 complex and mitochondrial damage may be a molecular mechanism for propofol-induced cell death.

PRIS can be lethal, and multiple studies suggest caution when using prolonged ( > 48 h) propofol sedation at doses higher than 5 mg/kg/h, particularly in patients with inflammatory illnesses^5,6,36^. The results from our findings demonstrate that propofol-induced pyroptosis not only triggers macrophage death but also mediates the release of IL-1β and IL-18, leading to an exaggerated proinflammatory process and multiple-organ dysfunction in the clinic. Therefore, inhibiting macrophage pyroptosis and consequent cytokine release would reduce the adverse effects of high-dose propofol administration. In addition, direct ultrastructural evidence for the central role of mitochondrial damage in patients receiving propofol infusion indicates impairment of mitochondrial function as the probable main cause of this syndrome^37^. Here, we provide in vitro evidence for profound mitochondrial damage as a correlate for increased mitochondrial ROS. As the management of PRIS remains difficult due to the rapid deterioration of patients with the disorder, suitable diagnostics and avoidance of other triggering factors (particularly in patients with acute neurological, inflammatory illnesses or mitochondrial diseases) remain a priority.

Collectively, our results indicate a previously unknown role of propofol in pyroptosis induction and the existence of an NLRP3/ASC/caspase-1 pathway in macrophages that contributes to cell damage. These findings further expand our understanding of the diverse functions of macrophages and provide potential targets for the treatment of PRIS.

## Methods

### Cell culture and propofol administration

DC2.4, BV2, H7C2, Hep1–6, THP-1, PBMCs and the mouse macrophage cell lines RAW264.7 and J774 were obtained from the ATCC. RAW-asc cells, a RAW264.7 cell line ectopically expressing ASC^15^, were kind gifts from Professor Jiahuai Han (Xiamen University, Xiamen, China). DC2.4, BV2, H7C2, Hep1–6, PBMCs, RAW264.7, RAW-asc and J774 cells were grown in Dulbecco’s modified Eagle’s medium (DMEM) supplemented with 10% fetal bovine serum (FBS). THP-1 was grown in RPMI-1640 medium with 10% FBS. All cells were cultured at 37 °C in a 5% CO_2_ incubator. Before the experiment, the cells were seeded in 6- or 96-well plates and incubated for 24 h.

WT C57BL/6J male mice were used to prepare BMDMs, as previously described^38^. First, mice were sacrificed by cervical dislocation, and then, femurs were harvested and cut with an amputation saw. Next, bone marrow cells from all bones were carefully flushed out. After centrifuging for 5 min at 310 × *g*, erythrocytes were eliminated using Red Blood Cell Lysing Buffer (Sigma-Aldrich, USA). The remaining cells were cultured in RPMI 1640 (10% FBS, 30 U/mL penicillin and 30 μg/mL streptomycin) supplemented with 50 mg/mL recombinant mouse M-CSF (R&D Systems, Minneapolis, MN, USA) for 7 days to allow for differentiation into BMDMs. On day 7, the macrophages were harvested, counted by automated cell counting (Nexcelom Bioscience, Lawrence, MA), and seeded into 6- or 96-well tissue culture plates for further study.

To evaluate caspase-1-induced pyroptotic cell, we first wanted to identify various cell lines that respond to propofol. The groups were as follows: a control group and propofol groups (6, 60 and 600 μM in medium).

The low-dose exposure concentrations in vitro correlated with the measured blood concentration of propofol in patients after intravenous administration of clinically relevant doses^12,39,40^. In addition, the reasoning for exposure to propofol at a high dose in vitro was that the concentration of propofol in tissue samples taken from an in vivo animal model of PRIS can reach a concentration of ~200 μM^7^. Thus, propofol dosages of 12, 60, 300 and 600 μM (corresponding to 0.5–10 times the clinical plasma concentration) were chosen for administration in macrophages. Propofol-enriched medium was prepared as described in a previous study^12^. For our in vitro studies here, propofol (Sigma-Aldrich, USA) was diluted in dimethyl sulfoxide (DMSO) to 600 mM and stored. The stock solution of propofol was diluted with medium to the desired concentrations to induce cell death. On the day of the experiment, the groups were as follows: a DMSO control group and propofol groups (12, 60, 300, and 600 μM in medium). Propofol-supplemented medium was then added to the cell cultures for 3 and 6 h. To explore the effects of caspase-1 inhibitors on propofol-induced cell death, VX-765 and Z-VAD-FMK (InvivoGen, San Diego, CA, USA) were dissolved in DMSO to give a final concentration of 50 mM and added to culture wells at a final concentration of 40 or 20 μM, respectively, 0.5 h before adding propofol. Following exposure, the supernatants and cells were harvested and used in a cell viability assay or immunoblot analysis.

Meanwhile, LPS-primed cells (50 ng/mL, 4 h) treated with 10 mM nigericin (InvivoGen, San Diego, CA, USA) for 2 h were used as a positive control for caspase-1-induced pyroptosis; J774 cells were treated with LPS (1 μg/mL, overnight; InvivoGen, San Diego, CA, USA) as a positive control for caspase-11 activation.

In addition, we incubated BMDMs and J774 cells with 300 μM propofol at different time points (0, 10, 20, 30, 60, 120 and 180 min) to further verify whether propofol is able to induce pyroptosis more rapidly than apoptosis.

### Animals and propofol injection

WT C57BL/6J male mice (2-months-old) were obtained from the Medical Laboratory Animal Centre of Guangdong Province (approval No. SCXK (Yue) 2008–0002), Foshan, China. Male NLRP3^-/-^ mice on the C57BL/6J genetic background were from the Model Animal Research Centre (AAALAC accredited, Nanjing University, China). All animals were allowed free access to food and water and were maintained under standard conditions (23 ± 1 °C, 55 ± 5% humidity, 12/12-h light–dark cycle). The mice were acclimatised for 1 week.

Two-month-old mice were placed in a temperature-controlled incubator (37 °C) and intraperitoneally administered propofol or 10% intralipid as a vehicle control. The aim of this experiment was to determine whether propofol causes pyroptosis and, if so, to establish the minimal effective dose of propofol for triggering a significant pyroptosis response. Most patients with PRIS have been sedated for a long time ( > 48 h) and with high doses ( > 5 mg/kg/h), and the human dosage was converted into an equivalent dosage appropriate for mice. Thus, WT mice (*n* = 8 per group) received a single intraperitoneal injection of vehicle or propofol at 0, 50, or 100 mg/kg for 7 consecutive days. Male NLRP3^-/-^ mice also received intraperitoneal injections of either 10% intralipid or 100 mg/kg/d propofol (Fresenius Kabi Deutschland GmBH, Germany) for 7 days. On day 7, mice were sacrificed 3 h after the last exposure, and blood and organs (spleen tissue) were collected. Serum and organs were stored at –80 °C until use. This dose of propofol was selected based on previous reports by other groups showing that sub-anaesthetic doses of 50 and 100 mg/kg propofol could trigger apoptosis^12^. All animal procedures were performed in strict accordance with the recommendations in the Guide for the Care and Use of Laboratory Animals of the National Institutes of Health (NIH). The protocol was approved by the Jinan University Institutional Animal Care and Use Committee (IACUC). All efforts were made to minimise both the suffering and the number of animals used.

### Cell viability assay

Cells were exposed to propofol, and the number of surviving cells was measured by a CCK-8 assay (Dojindo, Tokyo, Japan). Cells were plated at a density of 0.5 × 10^4^ cells/well in 96-well plates. The plate was pre-incubated for 12 h at 37 °C in a humidified 5% CO_2_ incubator. After exposure to propofol, 10 µL of CCK-8 solution was added to each well of the plate, and the plates were incubated for 0.5 h. Assay plates were shaken on an orbital shaker for 2 min and incubated at room temperature on the benchtop for 10 min. Data acquisition was then performed using a microplate reader (Spark 10 M, Tecan). Values were calculated according to the formula: cell viability (%) = [(As − Ab)/(Ac − Ab) × 100%, where As, Ac and Ab represent the absorbance at 450 nm (A450) in the treated, untreated and blank groups, respectively.

### LDH release assay

Cell death was measured by an LDH assay using a CytoTox 96 Non-Radioactive Cytotoxicity Assay kit following the manufacturer’s instructions (Promega). Data acquisition was then performed using a microplate reader (Spark 10 M, Tecan).

### Western blot analysis

For western blot analysis, the concentrated supernatants, adhered cells or spleens of mice were lysed, and the protein concentrations were measured as described previously^16^. An equivalent amount of each sample was separated by sodium dodecyl sulfate polyacrylamide gel electrophoresis (SDS-PAGE) and electrotransferred onto polyvinylidene fluoride membranes, which were blocked for 2 h at room temperature with 5% bovine serum albumin. The membrane was incubated with primary antibodies at 4 °C overnight. Then, the membrane was incubated with secondary antibodies for 1 h at room temperature, followed by visualisation using ECL reagent (Thermo Scientific, USA). Immunoblot detection was achieved by exposure with a chemiluminescence imaging system (ClinX Sciences instrument). The membranes were then stripped, reprobed with anti-GAPDH antibodies and exposed again to detect the endogenous GAPDH standard. ImageJ software was then used to scan and quantify the immunoblots. The band intensity values of the target proteins were normalised to that of GAPDH.

### Imaging of pyroptotic cell death by microscopy

To examine cell death morphology, cells were treated as indicated in six-well plates for image capture. Nuclei (red) were counterstained with PI (5 ng/mL), and cells were observed under a fluorescence microscope (Leica DMi8, Germany).

To identify pyroptotic cell death morphology, macrophages were plated as indicated in 24-well plates and treated with propofol (12, 60, 300 and 600 μM in medium). A FAM-FLICA Caspase-1 Detection Kit was used to detect active caspase-1 in macrophages. This kit employs the fluorescent inhibitor probe FAM-YVAD-FMK (FLICA) to label in situ active caspase-1 enzyme in living cells, and then, the fluorescent signal is analysed using fluorescence microscopy and a fluorescence plate reader. Nuclear staining was also performed with DAPI. According to the manufacturer’s instruction, pyroptotic cells were observed under a fluorescence microscope (Leica DMi8, Germany) for imaging in an optimal excitation range from 490 to 495 nm and optimal emission range from 515 to 525 nm. Images were processed using ImageJ software (NIH, Bethesda, MD, USA). The fluorescence intensity of cleaved caspase-1 was quantified by Image Pro Plus 6.0 software (Media Cybernetics, Silver Spring, MD, USA).

We also determined caspase-1 activity on frozen spleen sections using the FAM-FLICA Caspase-1 kit as previously described^41^. First, we prepared 7-µm-thick frozen spleen tissue sections and allowed the sections to air dry on slides (~10–15 min). Then, the slides were fixed with acetone for 2 min, rehydrated by washing (twice for 5 min) in TBS-Tween, and blocked for 20 min with blocking solution containing 20% Aqua-Block in medium with 0.2% Tween. We decanted the blocking solution and then applied 50 µL of 3 × FLICA working solution (freshly prepared by diluting 150 × stock solution 1:50 in phosphate-buffered saline (PBS)) per section and incubated the slides protected from light at room temperature for 2 h. Tissues were washed with Tris-buffered saline (TBS)-Tween (twice for 5 min). Finally, we mounted tissues with Vectashield mounting medium containing DAPI, and fluorescent images were captured with a fluorescence microscope.

### Caspase activation assay

Active caspase-1 and caspase-3/7 were quantified by using a FAM-FLICA detection kit (Immunochemistry Technologies, Bloomington, MN, USA) according to the manufacturer’s instructions. Samples were read on a 96-well fluorescence plate reader (Spark 10 M, Tecan).

### Flow cytometric analyses

After 20 min of propofol stimulation, BMDMs were incubated with MitoSOX (Invitrogen, Carlsbad, CA, USA) or MitoTracker Green and Deep Red (Invitrogen, Carlsbad, CA, USA). Mitochondrial mass was measured by fluorescence levels upon staining with MitoTracker Green and MitoTracker Deep Red at 100 nM for 30 min at 37 °C. Mitochondria-associated ROS levels were measured by staining cells with MitoSOX at 2.5 mM for 30 min at 37 °C. Cells were then washed with a PBS solution and resuspended in a cold PBS solution containing 1% FBS for fluorescence-activated cell sorting (FACS) analysis. Data were acquired on a FACSCalibur flow cytometer (BD Biosciences) and analysed with FlowJo (TreeStar).

### Generation of knockout cell lines using the *CRISPR/Cas9* technique

We knocked out caspase-1 in BMDMs using the clustered regularly interspaced short palindromic repeats (*CRISPR*)/*Cas9* method. Generation of knockout cell lines was accomplished as described^15^. The sequence in the genomic RNA (gRNA) vector was 3′-TCTCTAAAAAAGGGCCCC-5′ for mouse caspase-1. The plasmids harbouring the gene gRNA sequences and *Cas9* gene were transfected into cells in the presence of lentivirus helper plasmids, and the supernatants were collected after 48 h. Constructs were packaged into lentiviruses in HEK 293T cells in the presence of helper plasmids (pMDLg, pRSV-REWV and pVSV-G). The viruses were first concentrated and then used to infect BMDMs. To confirm allelic modifications, genomic DNA was harvested using a Blood and Tissue Culture Kit (QIAGEN, Valencia, CA, USA). A region containing the gRNA cut site was then PCR amplified and subcloned. Single-bacterial colonies were isolated and sequenced.

### Cytokine measurement

Cell-free culture supernatants were tested for IL-1α, TNF-α, IL-6 and MCP-1 concentrations by Milliplex assays (#HCYTOMAG-60K, Merck Millipore, USA) following the manufacturer’s protocols. Samples were analysed by a Luminex® 200™ System multiplex detection system (Luminex Corporation, Austin, TX, USA).

IL-1β and IL-18 levels in serum and supernatants were measured by enzyme-linked immunosorbent assay (ELISA) using a mouse IL-1β and IL-18 ELISA Kit (Abcam, Cambridge, MA, USA) according to the manufacturer’s instructions.

### Statistical analysis

All experiments were repeated at least three times independently, and the data are expressed as the mean ± standard error (SE). Data were analysed with analysis of variance (ANOVA) followed by Duncan’s test (SPSS, Inc., Cary, NC, USA). *P*-values below 0.05 were considered statistically significant.

## Supplementary information

Supplementary figure legends

Supplementary Figure 1

Supplementary Figure 2

Supplementary Figure 3

Supplementary Figure 4

Supplementary materials
