# Supplementary material for: Propofol directly induces caspase-1-dependent macrophage pyroptosis through the NLRP3-ASC inflammasome
Source: Cell Death Dis. 2019 Jul 17;10(8):542. doi: 10.1038/s41419-019-1761-4 (PMC6637184; doi:10.1038/s41419-019-1761-4)
Supplement: Supplementary file 1 — Supplementary figure legends [file 41419_2019_1761_MOESM1_ESM.docx]

**Supplemental Figure legends**

**Figure S1.** **Immunofluorescence staining of DAPI (blue) and cleaved-Caspase-1 (green) in J774 cells after 6 hours of exposure.** Representative microscopy images: Red arrows in magnified graph denote the fluorescence of cleaved-Caspase-1. Bars indicate scale of 50 and 15 μm, respectively.

**Figure S2. Propofol induces J774 cell death.** Immunoblots from J774 cells for the proteins associated with pyroptosis (A) and apoptosis (B). (C) Western blot analysis was used to determine the expression of inflammasomes and ASC. Band intensity were quantified by ImageJ software and the values of target proteins were normalized to that of GAPDH. Data are presented as mean ± SE (n = 3). Means of exposure not sharing a common letter are significantly different at p < 0.05 as assessed using one-way analysis of variance followed by the Duncan’s test. CASP1 = caspase-1; CASP3 = caspase-3; CASP7 = caspase-7; CASP8 = caspase-8; CASP9 = caspase-9; CASP11 = caspase-11; Sup = supernatant; Ext = extract.

**FIGURE S3.** **Time-dependent toxicity of propofol was analyzed by Western blotting.** J774 cells were treated with propofol (300 μM) or vehicle for the indicated time periods. (A) Immunoblots of cell extracts and supernatants from J774 cells for the proteins associated with pyroptosis. (B) Immunoblots from J774 cells for the proteins associated with apoptosis. (C) Western blot analysis was used to determine the expression of inflammasomes and ASC. Band intensity were quantified by ImageJ software and the values of target proteins were normalized to that of GAPDH. The data are expressed as mean ± SE (n = 3). Means of treatments not sharing a common letter are significantly different at P < 0.05 as assessed using one-way ANOVA followed by Duncan’s test. CASP1 = caspase-1; CASP3 = caspase-3; CASP7 = caspase-7; CASP9 = caspase-9; Sup = supernatant; ext = extract.

**FIGURE S4. Propofol induced apoptosis in splenic macrophages.** Western Blot analysis of apoptosis-related proteins. CASP3 = caspase-3; CASP7 = caspase-7; CASP9 = caspase-9.
