# Supplementary figures and images for: Propofol directly induces caspase-1-dependent macrophage pyroptosis through the NLRP3-ASC inflammasome
Source: Cell Death Dis. 2019 Jul 17;10(8):542. doi: 10.1038/s41419-019-1761-4 (PMC6637184; doi:10.1038/s41419-019-1761-4)

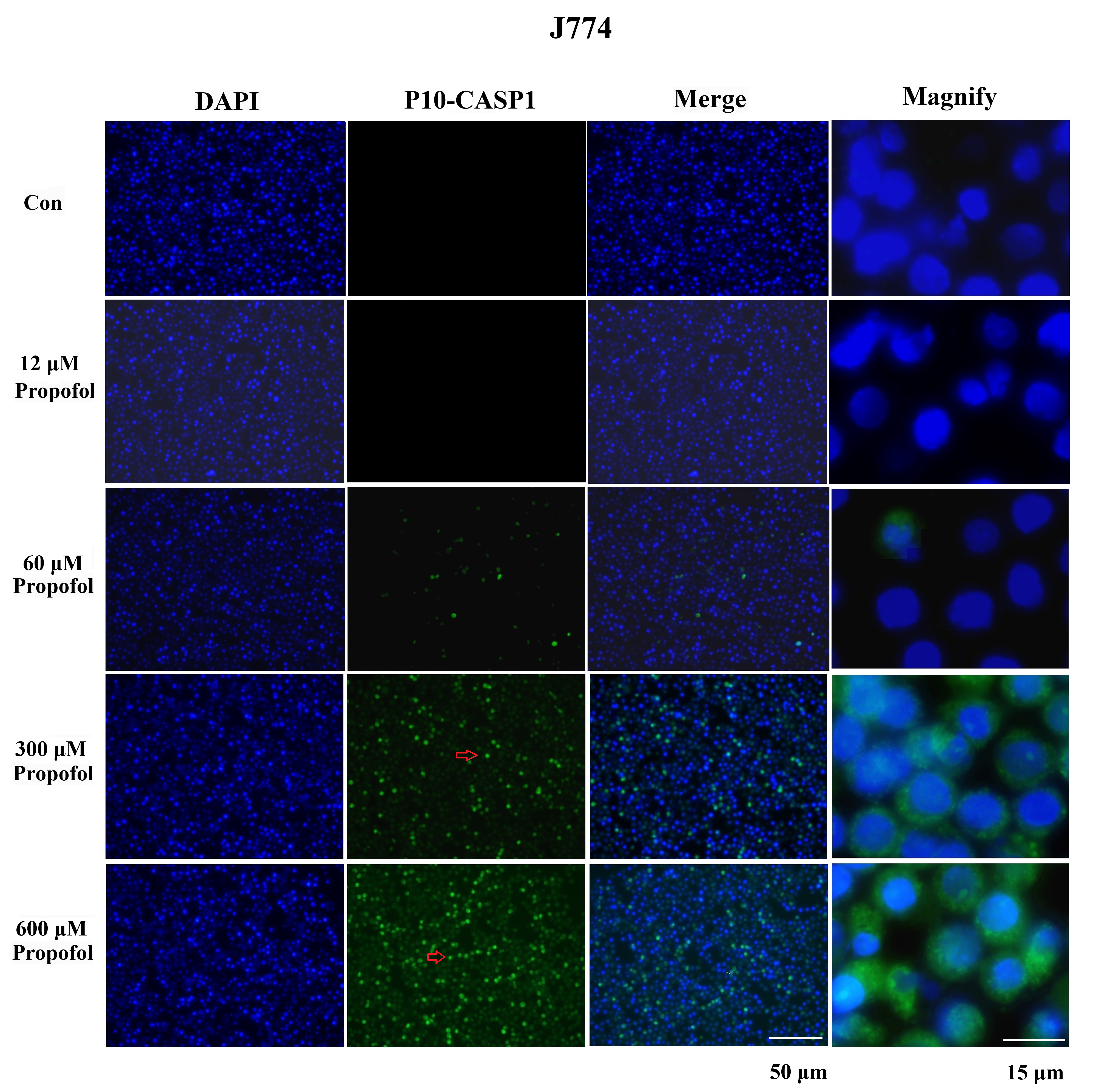

Supplement: Supplementary file 2 — Supplementary Figure 1 [file 41419_2019_1761_MOESM2_ESM.tif]

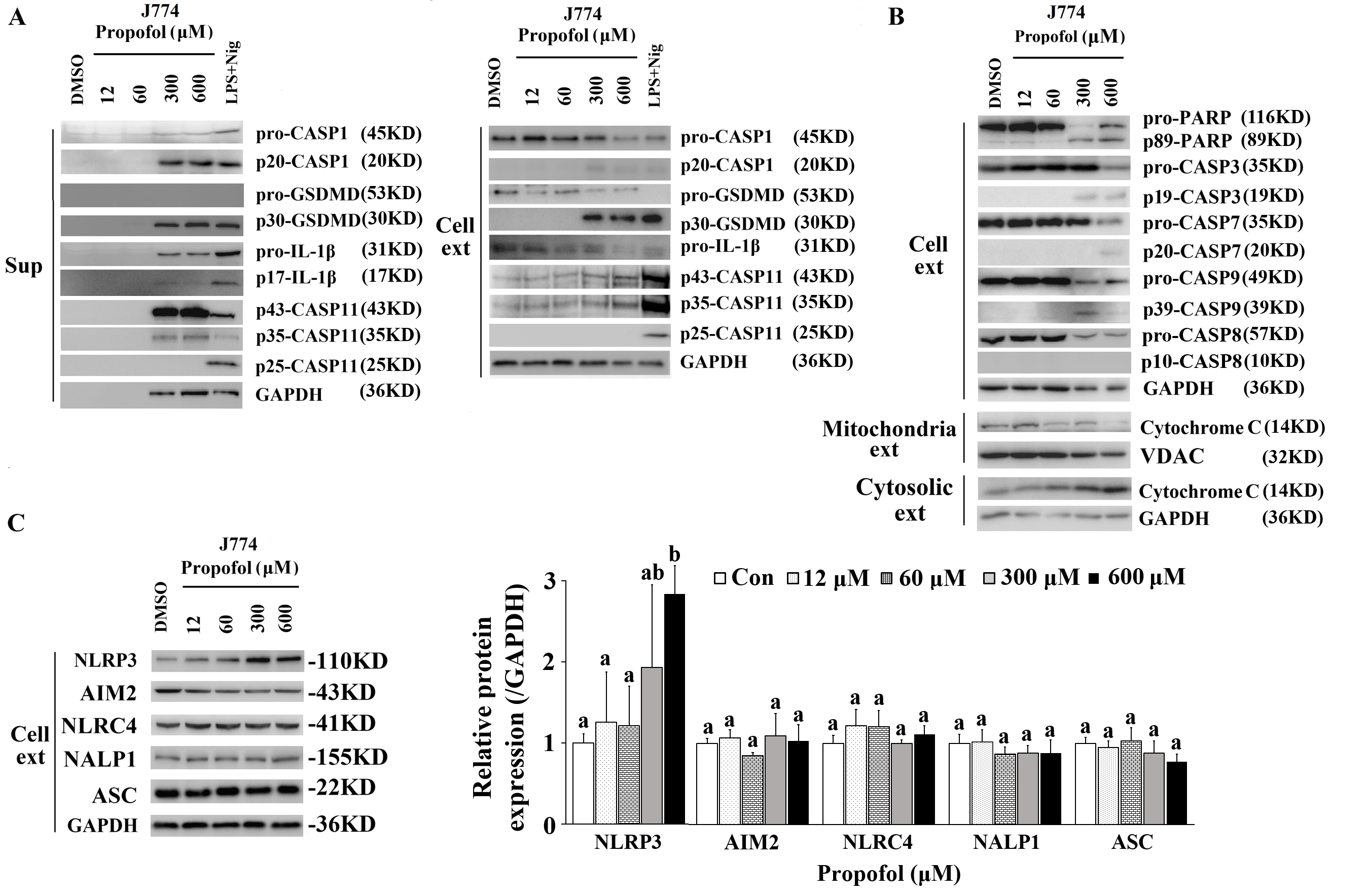

Supplement: Supplementary file 3 — Supplementary Figure 2 [file 41419_2019_1761_MOESM3_ESM.tif]

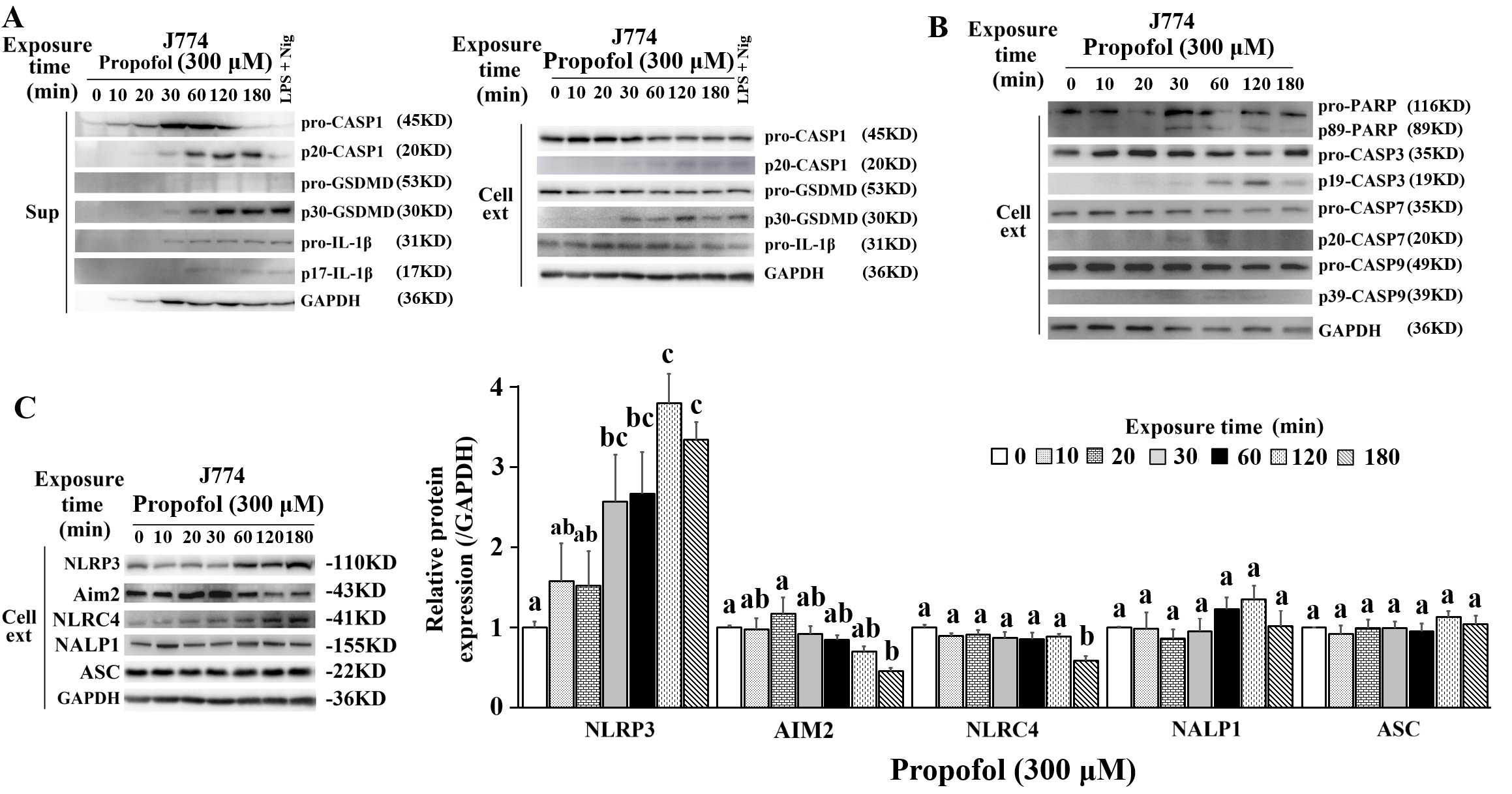

Supplement: Supplementary file 4 — Supplementary Figure 3 [file 41419_2019_1761_MOESM4_ESM.tif]

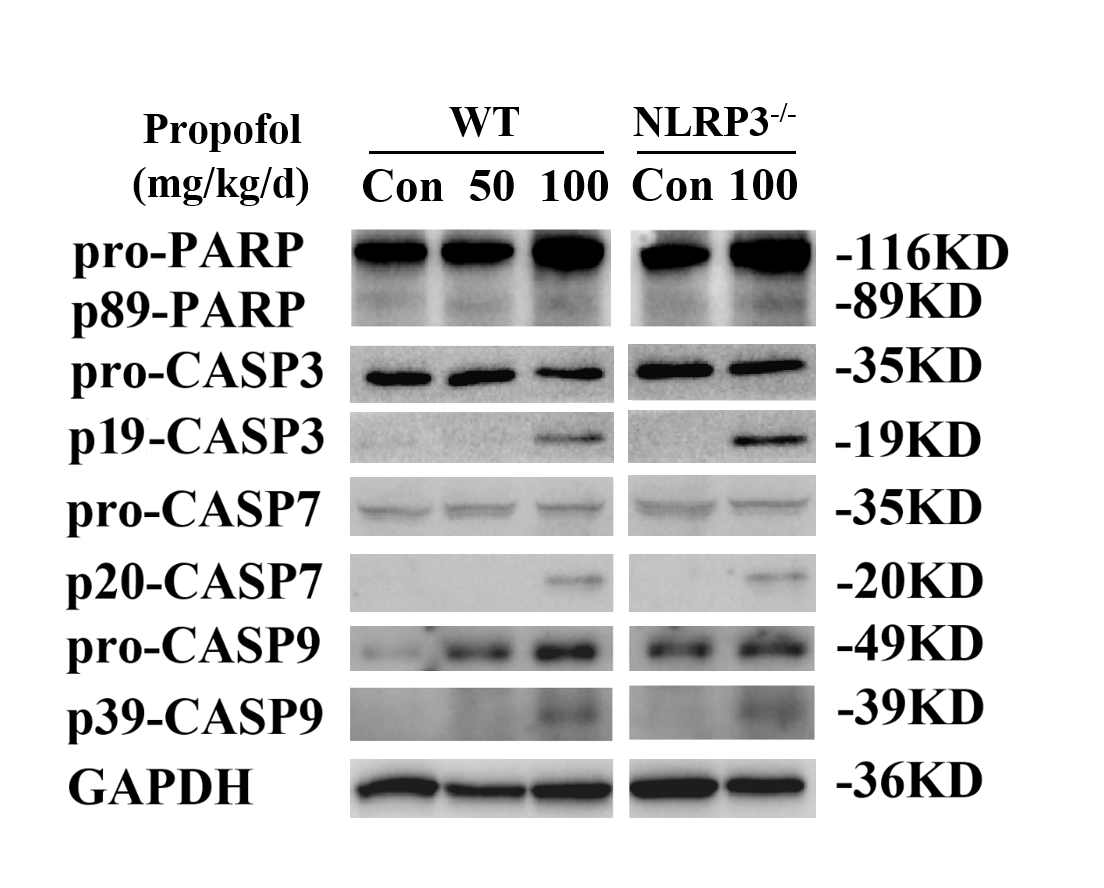

Supplement: Supplementary file 5 — Supplementary Figure 4 [file 41419_2019_1761_MOESM5_ESM.tif]
