# Supplementary material for: Propofol directly induces caspase-1-dependent macrophage pyroptosis through the NLRP3-ASC inflammasome
Source: Cell Death Dis. 2019 Jul 17;10(8):542. doi: 10.1038/s41419-019-1761-4 (PMC6637184; doi:10.1038/s41419-019-1761-4)
Supplement: Supplementary file 6 — Supplementary materials [file 41419_2019_1761_MOESM6_ESM.zip]

**Supporting Information**

**Supplemental Materials and Methods**

**Chemicals**

RIPA lysis buffer for protein extraction was purchased from Beyotime Biotechnology (Beyotime, China). The Pierce Lane Marker Reducing Sample Buffer, BCA protein assay kit and SuperSignal chemiluminescent substrate (ECL) were purchased from Thermo Scientific (Waltham, MA, USA). Red blood cell lysing buffer (R7757), PI (P4864) and DAPI (D9542) were purchased from Sigma-Aldrich. FAM-FLICA™ Caspases 1 kit (ICT097) and FAM-FLICA CASPASE-3/7 KIT (ICT093) were purchased from Immunochemistry Technologies (Bloomington, MN), recombinant mouse M-CSF from R&D systems, nigericin (#14K05-MM), VX-765 (inh-vx765i-1), Z-VAD-FMK (tlrl-vad) and Ultrapure LPS from *E. coli* O111:B4 (#13I06-MM) from InvivoGen Company (USA). CytoTox 96 Non-Radioactive Cytotoxicity Assay kit was purchased from Promega. A CCK-8 was purchased from Dojindo (Tokyo, Japan). The Mouse IL-1β ELISA kit (ab100704) and Mouse IL-18 ELISA kit (ab216165) were obtained from Abcam (Cambridge, MA, USA). QIAGEN DNeasy Blood & Tissue Kit was purchased from QIAGEN (Valencia, CA, USA). A commercially available multiplex immunoassay kit (Milliplex MAP kit; #HCYTOMAG-60K) was acquired from Merck Millipore (USA). MitoSOX, MitoTracker Green and Deep Red were purchased from Invitrogen (Carlsbad, CA, USA). For cell culture experiments, propofol (Sigma-Aldrich, USA) was stored at 4 °C in a polypropylene tube until the day of experiments. Intraperitoneally received propofol and 10% intralipid were purchased from Fresenius Kabi Deutschland GmbH, Germany. MACS CD11b^+^ MicroBeads was obtained from Miltenyi Biotec (Bergisch Gladbach, Germany).

**Antibodies**

Antibodies used include: IL-1β (5129-100, Biovision), mouse AIM2 (#13095, Cell Signaling Technology), NLRP3 (ab214185, Abcam), NALP1 (ab98181, Abcam), NLRC4 (#06-1125, Merck Millipore), GSDMD (ab209845, Abcam), Caspase-11 (AG-20T-0140-C100, Adipogen), Caspase-3 (9665, Cell Signaling Technology), Caspase-7 (12827, Cell Signaling Technology), Caspase-8(4927, Cell Signaling Technology), PARP (9532, Cell Signaling Technology), Cytochrome C (11940S, Cell Signaling Technology), mouse Caspase-9 (9504, Cell Signaling Technology), mouse apoptosis-associated speck-like protein (ASC) (#67824, Cell Signaling Technology), VDAC (#4866, Cell Signaling Technology) and mouse GAPDH (HC301-02, TransGen Biotech). Besides, antibody for Caspase-1 p20 (clone 4B4) was a kind gift from VM Dixit (Genetech, USA).

**Western blotting**

An equal number of cells were seeded per sample in order to ensure equal sample loading for all supernatants analysed by Western blot. Supernatant sample preparation: proteins from cell-free culture supernatants were concentrated using trichloroacetic acid (TCA). One hundred percent TCA was added to supernatants for a final concentration of 20%. Samples were vortexed and incubated on ice for 40 min. After incubation, samples were centrifuged at 800 × g and supernatants were discarded. The pellets were washed twice with 100% cold acetone and allowed to air-dry. Pellets were resuspended with Pierce Lane Marker Reducing Sample Buffer and boiled at 95°C for 10 min. Samples were loaded and run on SDS-PAGE gels

Cells or tissue lysates sample preparation: Cells or tissue lysates were obtained using RIPA Lysis. The lysates were centrifuged, and the supernatants were determined by BCA kit. Samples were resuspended with water and Pierce Lane Marker Reducing Sample Buffer and boiled at 95°C for 5 min. Samples were run and analysed as described in the preceding texts.

For western blot analysis, the concentrated supernatants, adhered cells or spleens of mice were lysed and the protein concentrations were measured and analysed. Samples were separated by 8 and 12% SDS-PAGE and then were transferred to PVDF membranes (Millipore). After blocking with 5% skimmed milk for 2 hours at room temperature, the membrane was incubated with primary antibodies to Caspase-7 (1:500), AIM2 (1:1000), IL-1β (1:1000), NLRP3 (1:1000), NALP1 (1:500), NLRC4 (1:500), GSDMD (1:2000), Caspase-11 (1:500), Caspase-3 (1:1000), Caspase-7 (1:1000), PARP (1:1000), Cytochrome C (1:2000), Caspase-9 (1:1000), VDAC (1:1000) and GAPDH (1:5000) at 4 °C overnight. Then the membrane was incubated with secondary antibodies for 1 hour at room temperature, followed by visualization using ECL reagent and the protein bands were obtained using a chemiluminescence detection system (ClinX Sciences instrument). ImageJ software was then used to scan and quantify the immunoblots. The band intensity values of the target proteins were normalized to that of GAPDH.

**Macrophage separation from spleen**

Mice splenocytes were harvested and washed in ice-cold RPMI 1640 medium (Invitrogen, Australia). Tissue was triturated with the sterile syringes, and the resulting cell suspension was filtered through 40-*μ*m nylon mesh and then incubated at 37°C for 30 min. The [adherent cells](https://www.sciencedirect.com/topics/medicine-and-dentistry/adherent-cell) were harvested and purified by MACS CD11b^+^ MicroBeads.

**Detailed Attribution of Authorship:**

L.S. designed the study, conducted the study and wrote the manuscript; W.M. conducted the study; W.G. analyzed the data; Y.X. analyzed the data; L.C. wrote the manuscript; Z.X. wrote the manuscript; Z.Z. wrote the manuscript; Z.D. analyzed the data. All authors read and approved the final manuscript.

In Figure 1, L.S. generated the immunofluorescence data and labelled the image; In Figure 2, L.S. generated the data and assembled the figure; In Figure 3, L.S. generated the data and assembled the figure; In Figure 4, L.S. generated the data and assembled the figure; In Figure 5, L.S. generated the data and assembled the figure; In Figure 6, L.S. generated the data and assembled the figure; In Figure 7, L.S. generated the data and assembled the figure; In Figure 8, L.S. generated the the image.
